# A Review of Malaysian Medicinal Plants with Potential Anti-Inflammatory Activity

**DOI:** 10.1155/2018/8603602

**Published:** 2018-07-09

**Authors:** Fazleen Izzany Abu Bakar, Mohd Fadzelly Abu Bakar, Norazlin Abdullah, Susi Endrini, Asmah Rahmat

**Affiliations:** ^1^Faculty of Applied Sciences and Technology, Universiti Tun Hussein Onn Malaysia (UTHM), Hab Pendidikan Tinggi Pagoh, KM 1, Jalan Panchor, 84600 Muar, Johor, Malaysia; ^2^Centre of Research for Sustainable Uses of Natural Resources (CoR-SUNR), Universiti Tun Hussein Onn Malaysia (UTHM), Parit Raja, 86400 Batu Pahat, Johor, Malaysia; ^3^Faculty of Medicine, YARSI University, 10510 Jakarta, Indonesia

## Abstract

This article aims to provide detailed information on Malaysian plants used for treating inflammation. An extensive search on electronic databases including PubMed, Google Scholar, Scopus, and ScienceDirect and conference papers was done to find relevant articles on anti-inflammatory activity of Malaysian medicinal plants. The keyword search terms used were “inflammation,” “Malaysia,” “medicinal plants,” “mechanisms,” “*in vitro*,” and “*in vivo*.” As a result, 96 articles on anti-inflammatory activity of Malaysian medicinal plants were found and further reviewed. Forty-six (46) plants (*in vitro*) and 30 plants (*in vivo*) have been identified to possess anti-inflammatory activity where two plants, *Melicope ptelefolia* (Tenggek burung) and *Portulaca oleracea* (Gelang pasir), were reported to have the strongest anti-inflammatory activity of more than 90% at a concentration of 250 *µ*g/ml. It was showed that the activity was mainly due to the occurrence of diverse naturally occurring phytochemicals from diverse groups such as flavonoids, coumarins, alkaloids, steroids, benzophenone, triterpenoids, curcuminoids, and cinnamic acid. Hence, this current review is a detailed discussion on the potential of Malaysian medicinal plants as an anti-inflammatory agent from the previous studies. However, further investigation on the possible underlying mechanisms and isolation of active compounds still remains to be investigated.

## 1. Introduction

A primary physiologic defence mechanism known as inflammation helps to protect the body from noxious stimuli, resulting in the swelling or edema of tissues, pain, or even cell damage. The main purpose of this mechanism is to repair and return the damaged tissue to the healthy state [1]. The increase in size of the vessels only occurs around the inflammatory loci (i.e., neutrophils, macrophages, and lymphocytes) during the early stages of inflammation, but after 24 hours, many kinds of cells reach neutrophils, followed by macrophages within 48 hours and lymphocytes after several days [1]. It is well known that the disruption of cells occurs during inflammation processes, leading to the release of arachidonic acid, and further undergoes two metabolic pathways known as the cyclooxygenase (COX) and lipoxygenase (LOX) pathways. COX pathways consist of cyclooxygenase-1 (COX-1) and cyclooxygenase-2 (COX-2), while 5-lipoxygenase (5-LOX), 12-lipoxygenase (12-LOX), and 15-lipoxygenase (15-LOX) are the examples of the LOX pathway. The products of the COX pathway are prostaglandins (mediators of acute inflammation) and thromboxanes, while those of the LOX pathway are leukotrienes and hydroperoxy fatty acids [2, 3].

Clinically, the common signs of inflammation include pain, heat, redness, loss of function, and swelling on the affected tissue [4]. Other signs include fever, leukocytosis, and sepsis. There are many causes of inflammation such as pathogens (e.g., bacteria, viruses, and fungi), external injuries, and effects of chemicals or radiation. Inflammation can be classified into two categories: acute and chronic inflammation. Acute inflammation is considered as the first line of defence against injury. It occurs in a short period of time and is manifested by the excretion of fluid and plasma proteins along with the emigration of leukocytes such as neutrophils. Meanwhile, chronic inflammation takes prolonged duration and is manifested by the action of lymphocytes and macrophages, resulting in fibrosis and tissue necrosis. Inflammation is considered as one of the most common concern of diseases, ranging from the minor to a serious condition like cancer. Based on the recent advancement in imaging technologies, the chronic vascular inflammation is not involved in atherosclerosis but also in arterial hypertension and metabolic syndrome [5].

Currently, nonsteroidal anti-inflammatory drugs (NSAIDs) such as ibuprofen, aspirin, diclofenac, and celecoxib are extensively used for the treatment of inflammation. These drugs exhibit their anti-inflammatory properties by inhibiting the COX-1 activity and thus preventing the synthesis of prostaglandins [4]. However, the major concern is that NSAIDs may cause various side effects such as gastrointestinal complications [6]. Considering this, the quests for the new drug with anti-inflammatory properties from the medicinal plants with free of or fewer side effects are greatly needed for the pharmaceutical industry [7, 8].

Plant-based or herbal medicine has been used traditionally to treat pain, inflammation, and inflammatory-mediated pain [9]. Malaysia is among the world's 12 megadiverse countries where endemism is highest. At least a quarter of our tree flora is not found elsewhere in the world, and many of our herbaceous flora and other groups of species are unique [10]. In Malaysia, about 2000 medicinal plant species are reported to possess health benefit properties [11]. Based on nutritional studies, these medicinal plants contain diverse nutritive values and possess potential bioactive compounds with the activity related to the various inflammation disorders including gout [12] or age-related diseases [13]. Hence, this current review aims to disseminate detailed information on the anti-inflammatory potential of Malaysian medicinal plants, focusing on the bioactive phytochemicals, and mechanisms of action against inflammation in both *in vitro* and *in vivo* studies.

## 2. Methods

The bibliographic research was performed in the following databases: PubMed, Google Scholar, Scopus, and ScienceDirect, where these databases were searched for relevant studies which included at least one keyword from each of the following: (i) inflammation, (ii) Malaysia, (iii) medicinal plants, (iv) mechanisms, (v) *in vitro*, and (vi) *in vivo*. No limit was placed on the search time frame in order to retrieve all relevant papers, and the last search was performed on April 20, 2018. About 96 papers have been reviewed including journal articles and proceedings as well as the reference lists of articles for additional relevant studies.

## 3. Discussion

The World Health Organization (WHO) defines medicinal plants as plants which possess compounds that can be used for the therapeutic purposes as well as producing useful drugs from the metabolites. According to the WHO, medicinal plants are still being used by the people in developing countries to treat various diseases, and these products' market continue to grow [14] which gives a good sign of economic importance of medicinal plants. Based on the previous report, 15% out of 300,000 plant species in the world have been studied for the pharmacological activity. Interestingly, about 25% of modern medicines have been developed from the natural resources such as medicinal plants [15]. Recently, the research on the medicinal plants for the health benefit purposes has increased worldwide and gained attention from all researchers all over the world including Malaysia. Malaysia is known as a country that is rich in the medicinal plant species. For instance, 1300 medicinal plant species and 7411 plant species have been recorded in Peninsular Malaysia and Sabah, respectively [16, 17].

Inflammation is a response of tissue to cell injury due to pathogens, damaged tissues, or irritants which initiates the chemical signals to heal the afflicted tissue [18]. The leukocytes such as neutrophils, monocytes, and eosinophils are activated and migrated to the sites of damage. During the inflammatory processes, the excessive nitric oxide (NO) and prostaglandin E_2_ (PGE_2_) as well as proinflammatory cytokines (i.e., tumour necrosis factor-alpha (TNF-*α*) and interleukins) are secreted by the activated macrophages. The nitric oxide and prostaglandin productions from the inducible nitric oxide synthase (iNOS) and COX-2, respectively, are the proinflammatory mediators responsible for many inflammatory diseases [19, 20]. Inflammation can be classified into two types known as acute and chronic inflammation. The vascular response to inflammation in the early stage (acute inflammation) can be clearly seen at the affected tissue as it becomes reddened due to the increase of blood flow and swollen due to edema fluid. Three main processes that involve during the vascular response to acute inflammation are (1) changes in vessel caliber and blood flow, (2) the increase in vascular permeability, and (3) fluid exudate formation. It is important to understand that an uncontrolled inflammation may contribute to many chronic illnesses [21]. For instance, chronic inflammation may lead to infectious diseases and cancer [22], while the prolonged inflammation may cause abnormal gene expression, genomic instability, and neoplasia [23, 24]. Currently, NSAIDs exhibit great effects in inhibiting the activity of COX-1 and COX-2, but COX-1 inhibitors are reported to exert side effects such as gastrointestinal erosions and renal and hepatic insufficiency [25]. COX-2 (Vioxx) also has been reported to cause serious cardiovascular events [2]. To overcome this, many studies on anti-inflammatory drugs from natural resources have been conducted. Enzyme inhibitory assays (i.e., COX and LOX) have been extensively used to study the effectiveness of medicinal plants in treating the inflammation due to the presence of many phytochemicals, and they are being consumed as a food or food supplement for many years. The Malaysian medicinal plants that possess an anti-inflammatory activity are shown in Tables 1 and 2 for *in vitro* and *in vivo* studies, respectively.

Based on the results obtained, many studies used the NO inhibition assay as a method to show the anti-inflammatory activity of the plants. Many diseases such as rheumatoid arthritis, diabetes, and hypertension have been reported to be occurred due to the excessive production of NO [77]. NO is synthesized by inducible NO synthase which has three isomers: (i) neuronal nitric oxide synthase (nNOS), (ii) endothelial nitric oxide synthase (eNOS), and (iii) iNOS [78]. For instance, signaling molecules such as mitogen-activated protein kinases (MAPKs), nuclear factor-kappa B (NF-*κ*B), activator protein-1 (AP-1), and signal transducer and activator of transcription (STAT) regulate the inducible enzyme (i.e., iNOS), which then make this enzyme to be expressed in some tissues [79]. Apart from the nitric oxide inhibition assay, some studies used the LOX assay in order to evaluate the anti-inflammatory of the plants. In this mechanism, arachidonic acid is metabolized by 5-LOX to various forms of inflammatory leukotrienes such as leukotriene (LT) A_4,_ LTB_4,_ LTC_4_, LTD_4_, and LTE_4_ [80], where LTB_4_ (one of the mediators of inflammation) is reported to be the most crucial in the inflammatory response [81]. To support this, it is reported that patients with rheumatoid arthritis and inflammatory bowel disease possess high levels of LTB_4_ [82, 83]. In addition, LTs are reported to be linked with few diseases such as bronchial asthma and skin inflammatory disorders [84]. In 2011, Kwon et al. [85] demonstrated that esculetin, one of the examples of coumarins, exhibited anti-inflammatory activity *in vivo* against animal models of skin inflammation. In the LOX assay, any LOX inhibitors will reduce Fe^3+^ to Fe^2+^, providing a rapid colorimetric assay [26]. Another common assay in determining the anti-inflammatory activity is COX. Two isoforms of COX, COX-1 (mainly involved in physiological functions and constitutively expressed) and COX-2 (involved in inflammation and induced in the inflamed tissue), are the enzymes responsible for the synthesis of prostaglandins [86]. Besides, the COX-2 gene is also a gene for iNOS induced during inflammation and cell growth [87]. The Griess assay is another assay commonly used in the murine macrophage cell line (RAW 264.7) as a culture medium in the cell-based study in order to determine the concentration of nitrite (NO_2_−), the stable metabolite of NO.

Based on Table 1 (*in vitro* study), 46 plants have been identified and studied for the anti-inflammatory activity from the previous studies. As a result, only two plants have been reported to exhibit more than 90% of anti-inflammatory activity using the nitric oxide inhibition assay, which were *Melicope ptelefolia* (Tenggek burung) and *Portulaca oleracea* (Gelang pasir) with the values of 95.00% and 94.80% at 250 *µ*g/ml, respectively [13]. Besides that, many previous studies had reported the plants which exerted anti-inflammatory activity between 70% and 80% at 100 *µ*g/ml to 2000 *µ*g/ml which can be considered to be higher such as *Jatropha curcas* (Jarak pagar), *Curcuma longa* (Kunyit), *Boswellia serrata* (Kemenyan), *Labisia pumila* (Kacip fatimah), *Oenanthe javanica* (Selom), *Carica papaya* (Betik), and *Eurycoma longifolia* (Tongkat ali) with the values of 86.00%, 82.50%, 80.00%, 75.68%, 75.64%, 72.63%, and 70.97%, respectively [29, 31, 35, 36, 40, 42]. The moderate result of anti-inflammatory activity (50%–60%) also had been showed by several plants such as *Phaleria macrocarpa* (69.50%), *Sauropus androgynus* (68.28%), *Piper sarmentosum* (62.82%), *Thymus vulgaris* (62.00%), *Barringtonia racemosa* (57.70%), and *Kaempferia galanga* (57.82%) at 100 *µ*g/ml to 2000 *µ*g/ml [27, 31, 41, 43, 48]. In addition, plants from the Zingiberaceae, Lamiaceae, Annonaceae, and Fabaceae families have been studied extensively for the anti-inflammatory activity. Among these families, the active compound of *Curcuma longa* from the Zingiberaceae family, monodemethoxycurcumin, had the highest activity with 82.50% at 125 *µ*g/ml [35]. Of the other study, *Kaempferia galanga* from the Zingiberaceae family exhibited moderate activity with 57.82% at 200 *µ*g/ml where the isolated compound, ethyl-*p*-methoxycinnamate, was found to have anti-inflammatory activity via inhibiting the actions of COX-1 and COX-2 [41]. In the Lamiaceae family, *Thymus vulgaris* showed the highest percentage of anti-inflammatory activity compared to other plants with 62% at 100 *µ*g/ml [43], with the total phenolic content of 350 *µ*g GAE/ml.

In this study, it was found that the results of anti-inflammatory activity of the methanolic extract of the leaves of *Melicope ptelefolia* (Tenggek burung) varied between two previous studies due to the different types of assays used by both studies: nitric oxide inhibition and soybean 15-lipoxygenase inhibition assays with the values of 95% and 72.3%, respectively [13, 45]. Another study also reported that the anti-inflammatory activity of the methanolic extract of *Litsea garciae* fruits showed 9.42% (lipoxygenase assay) and 27.70% (hyaluronidase assay) [44]. Based on these results, it can be concluded that different assays used might produce different results. For the COX inhibition assay, all the curcumins isolated from *Curcuma longa* rhizomes (i.e., curcumin I, curcumin II (monodemethoxycurcumin), and curcumin III (bisdemethoxycurcumin)) displayed greater inhibition of COX-2 compared to COX-1 at the same test concentration [35]. For the Griess assay, all the species tested such as the leaves of *Carica papaya*, *Sauropus androgynus*, and *Piper sarmentosum*, the flowering stalk of *Musa acuminata*, and the whole plant of *Oenanthe javanica* displayed significant NO inhibitory activity in a concentration-dependent manner against IFN-*γ*/LPS-treated macrophages [31].

For the *in vivo* study (Table 2), 30 plants have been identified in this study for the anti-inflammatory activity. Many of the studies from the previous years used the carrageenan-induced rat paw edema method (a reliable inflammation model) as this carrageenan has been found to be more trenchant in producing the edema compared to formalin [88]. It is also one of the conventional methods used to evaluate the anti-inflammatory effect of drugs or medicinal plants at the acute stage [89] and involves a biphasic event. Normally, the release of histamine and serotonin happens in the early phase (1-2 h), while the second phase (3–5 h) involves the release of prostaglandins and kinins [90, 91]. For the edema formation, the rat paw is injected with carrageenan. This method is also a COX-dependent reaction with the control of arachidonate COX [92]. The ability of the plant extracts to lessen the thickness of the rats' paw edema indicates the ability of these plant extracts to exert the anti-inflammatory properties. Based on Table 2, the highest dose of the extract used was 1000 mg/kg of body weight, while the lowest one was 3 mg/kg of body weight. Most of the previous studies reported that the extract was able to inhibit paw edema induced by carrageenan. For instance, a significant highest paw edema inhibition (93.34%) was observed in rats at a dose of 300 mg/kg of the *Ardisia crispa* (Mata pelandok) root extract [51]. Another study also showed that a significant highest inhibition was observed in two isolated compounds from *Sandoricum koetjape* stems, 3-oxo-12-oleanen-29-oic acid and katonic acid with 94% and 81%, respectively, where 3-oxo-olean-12-en-29-oic acid had the percentage inhibition almost similar to the reference drug, indomethacin (97%) [71].

Based on the results obtained, few studies isolated the bioactive compounds to be further analyzed for the anti-inflammatory activity such as flavonoids (boesenbergin A, eupatorin, and sinensetin), coumarins (scopoletin and scoparone), triterpenoids (dammara-20,24-dien-3-one and 24-hydroxydammara-20,25-dien-3-one), steroids (cucurbitacin E), curcuminoids (monodemethoxycurcumin and bisdemethoxycurcumin), benzophenones (garsubellin A and garcinielliptin oxide), cinnamic acid (ethyl-*p*-methoxycinnamate), alkaloids (kokusaginine), benzene (*p*-*O*-geranylcoumaric acid), 4-[(20-*O*-acetyl-*α*-L-rhamnosyloxy)benzyl]isothiocyanate, 4-[(30-*O*-acetyl-*α*-L-rhamnosyloxy)benzyl]isothiocyanate, and 4-[(40-*O*-acetyl-*α*-L-rhamnosyloxy)benzyl]isothiocyanate [28, 30, 32, 33, 35, 38, 41, 45–47]. Interestingly, some of them exerted significant inhibition on inflammation. In 2000, Abad et al. [93] evaluated the common anti-inflammatory drug naproxene isolated from *Musa acuminate* (pisang abu nipah) which exhibited good inhibition in COX-1 and COX-2 activities. Besides, in *Carica papaya* leaves, coumarin was isolated and exerted anti-inflammatory activity by suppressing the cytokine TNF-*α* production [94, 95]. A compound known as dammara-20,24-dien-3-one was isolated from *Chisocheton polyandrus* and displayed good inhibition of both human 5-LOX and COX-2 [32]. Flavonoids have been confirmed by *in vitro* studies to be able to suppress iNOS expression and to prevent nitric oxide production, depending on their structure or subclass of flavonoids for the strength level [96].

## 4. Conclusion

In overall, this review clearly demonstrates the potential of Malaysian medicinal plants as anti-inflammatory agents in which *Melicope ptelefolia* (Tenggek burung) and *Portulaca oleracea* (Gelang pasir) were found to exhibit potent anti-inflammatory activity *in vitro*. Pharmacological studies revealed that chemical diverse groups of naturally occurring substances derived from the plants show promising anti-inflammatory activity. Therefore, this review suggests further research needs to be carried out on the bioactive compounds present in the particular plants which have a potential to treat an inflammation and the possible underlying mechanisms of inflammation.

## Figures and Tables

**Table 1:** The medicinal plants which are considered to possess anti-inflammatory activity based on *in vitro* studies.

Scientific name	Family	Local name	Part/solvent used	Types of assays	Anti-inflammatory activity (%)	IC_50_	Active compounds	References
*Agelaea borneensis*	Connaraceae	Akar rusa-rusa	Bark/methanol	LOX inhibition	71%–100% at 100 *μ*g/ml	NA	NA	[26]
*Anacardium occidentale*	Anacardiaceae	Pokok gajus	Leaves/methanol	NO inhibition	16.10% at 250 *μ*g/ml	NA	NA	[13]
*Averrhoa bilimbi*	Oxalidaceae	Belimbing buluh	Fruits/water	NO inhibition	22.30% at 250 *μ*g/ml	NA	NA	[13]
*Barringtonia racemosa*	Lecythidaceae	Putat kampung	Leaves/chloroform	Griess assay (NO inhibition)	57.7% at 100 *μ*g/ml	NA	NA	[27]
			Leaves/ethanol		29.80% at 100 *μ*g/ml			
			Leaves/hexane		42.39% at 100 *μ*g/ml			
*Boesenbergia rotunda*	Zingiberaceae	Temu kunci	Rhizomes/hexane	Griess assay (nitrite determination)	NA	36.68 *μ*M	Boesenbergin A	[28]
*Boswellia serrata*	Burseraceae	Salai guggul and kemenyan	Leaves/methanol	Human red blood cell method	80.00% at 2000 *μ*g/ml	NA	NA	[29]
*Buchanania insignis*	Anacardiaceae	Tais/mangga hutan	Bark/methanol	LOX inhibition	41%–70% at 100 *μ*g/ml	NA	NA	[26]
*Canarium patentinervium*	Burseraceae	Kedondong and kaju kedapak	Leaves and barks/hexane, chloroform, and ethanol	5-LOX inhibition	NA	1.76 *μ*M	Scopoletin	[30]
*Carica papaya*	Caricaceae	Betik	Leaves/methanol	Griess assay (NO inhibition)	72.63% at 100 *μ*g/ml	60.18 *μ*g/ml	NA	[31]
*Chisocheton polyandrus*	Meliaceae	Lisi-lisi	Bark/methanol	LOX inhibition	71%–100% at 100 *μ*g/ml	NA	NA	[26]
			Leaves/hexane, dichloromethane, and methanol	Soybean LOX inhibition assay	NA	0.69 *μ*M and 1.11 *μ*M	Dammara-20,24-dien-3-one and 24-hydroxydammara-20,25-dien-3-one	[32]
*Citrullus lanatus*	Cucurbitaceae	Tembikai	Fruit pulp/petroleum ether, chloroform, and 90% ethanol	COX-2 inhibitory activity	60–70% at 100 *μ*M	69 *μ*M	Cucurbitacin E	[33]
				Griess assay (NO inhibition)		17.6 *μ*M		
*Cosmos caudatus*	Asteraceae	Ulam raja	Leaves/methanol	NO inhibition	15.40% at 250 *μ*g/ml	NA	NA	[13]
*Crinum asiaticum*	Amaryllidaceae	Pokok bakung	Leaves/ethanol	NO inhibition	NA	58.5 *μ*g/ml	NA	[34]
*Curcuma longa*	Zingiberaceae	Kunyit	Rhizomes/hexane-ethyl acetate and methanol	COX-2 inhibitory activity	82.50% and 58.90% at 125 *μ*g/ml	NA	Monodemethoxycurcumin and bisdemethoxycurcumin	[35]
*Curcuma mangga*	Zingiberaceae	Temu mangga	Rhizomes/methanol	NO inhibition	19.20% at 250 *μ*g/ml	NA	NA	[13]
*Cythostema excelsia*	Annonaceae	Lianas	Leaves and stems/methanol	LOX inhibition	41%–70% at 100 *μ*g/ml	NA	NA	[26]
*Desmos chinensis*	Annonaceae	Kenanga hutan	Bark/methanol	LOX inhibition	41%–70% at 100 *μ*g/ml	NA	NA	[26]
*Eurycoma longifolia*	Simaroubaceae	Tongkat ali	Root/hydroalcoholics	Human red blood cell membrane stabilization method	70.97% at 1000 *μ*g/ml	NA	NA	[36]
*Ficus deltoidea*	Moraceae	Mas cotek	Leaves/methanol	LOX inhibition	10.35% at 100 *μ*g/ml	NA	NA	[37]
*Garcinia cuspidata*	Clusiaceae	Asam kandis	Bark/methanol	LOX inhibition	71%–100% at 100 *μ*g/ml	28.3 *μ*g/ml	NA	[26]
*Garcinia subelliptica*	Guttiferae	Pokok penanti	Seeds/chloroform	Chemical mediator released from mast cell and neutrophil inhibition	NA	15.6 *μ*M, 18.2 *μ*M, and 20.0 *μ*M	Garsubellin A and garcinielliptin oxide	[38]
*Gynura pseudochina*	Asteraceae	Pokok daun dewa	Leaves/ethyl acetate	IL-6/luciferase assay	NA	11.63 *μ*g/ml	NA	[39]
*Jatropha curcas*	Euphorbiaceae	Jarak pagar	Latex and leaves/aqueous methanol	NO inhibition	NA	29.7 and 93.5 *μ*g/ml	NA	[40]
*Kaempferia galanga*	Zingiberaceae	Cekur	Rhizomes/petroleum ether, chloroform, methanol, and water	COX-2 inhibitory screening assay	57.82% at 200 *μ*g/ml	0.83 *μ*M	Ethyl-*p*-methoxycinnamate	[41]
*Labisia pumila* var. alata	Myrsinaceae	Kacip fatimah	Roots/methanol	Colorimetric nitric oxide assay (macrophage cell line)	75.68% at 100 *μ*g/ml	NA	NA	[42]
*Leucas linifolia*	Lamiaceae	Ketumbak	Whole plant/methanol	LOX inhibition	34% at 100 *μ*g/ml	NA	NA	[43]
*Litsea garciae*	Lauraceae	Engkala/pengalaban	Fruits/methanol	LOX assay	9.42% at 2 mg/ml	NA	NA	[44]
				Hyaluronidase assay	27.70% at 5 mg/ml			
*Melicope ptelefolia*	Rutaceae	Tenggek burung	Leaves/methanol	NO inhibition	95% at 250 *μ*g/ml	NA	*p*-*O*-geranylcoumaric acid, kokusaginine, and scoparone	[13, 45]
				Soybean 15-LOX inhibition assay	72.3%			
*Moringa oleifera*	Moringaceae	Kelur	Fruits/ethyl acetate	NO inhibition	NA	0.136 *μ*g/ml 1.67 *μ*M	(1) 4-[(20-*O*-Acetyl-*α*-L rhamnosyloxy)benzyl]isothiocyanate	[46]
						2.66 *μ*M	(2) 4-[(30-*O*-Acetyl-*α*-L-rhamnosyloxy)benzyl]isothiocyanate	
						2.71 *μ*M	(3) 4-[(40-*O*-Acetyl-*α*-L-rhamnosyloxy)benzyl]isothiocyanate	
*Musa acuminata*	Musaceae	Pisang abu nipah	Flowering stalk/methanol	Griess assay (NO inhibition)	71.06% at 100 *μ*g/ml	42.24 *μ*g/ml	NA	[31]
*Ocimum basilicum*	Lamiaceae	Daun selasih	Leaves/methanol	NO inhibition	30.00% at 250 *μ*g/ml	NA	NA	[13]
*Ocimum canum*	Lamiaceae	Kemangi putih	Whole plant/methanol	LOX inhibition	32% at 100 *μ*g/ml	NA	NA	[43]
*Oenanthe javanica*	Apiaceae	Selom	Whole plant/methanol	Griess assay (NO inhibition)	75.64% at 100 *μ*g/ml	54.12 *μ*g/ml	NA	[31]
*Orthosiphon stamineus*	Lamiaceae	Misai kucing	Leaves/petroleum ether, chloroform, and methanol	NO inhibition	NA	5.2 *μ*M (eupatorin) 9.2 *μ*M (sinensetin)	Eupatorin and sinensetin	[47]
*Pandanus amaryllifolius*	Pandanaceae	Pandan	Leaves/methanol	NO inhibition	34.10% at 250 *μ*g/ml	NA	NA	[13]
*Persicaria tenella*	Polygonaceae	Daun kesum	Leaves/methanol	NO inhibition	87.80% at 250 *μ*g/ml	8 *μ*g/ml	NA	[13]
*Phaleria macrocarpa*	Thymelaeaceae	Mahkota dewa	Mesocarp/methanol	NO inhibition	69.50% at 200 *μ*g/ml	NA	NA	[48]
			Pericarp/methanol		63.40% at 200 *μ*g/ml			
			Seeds/methanol		38.10% at 200 *μ*g/ml			
*Piper sarmentosum*	Piperaceae	Kaduk	Leaves/methanol	Griess assay (NO inhibition)	62.82% at 100 *μ*g/ml	60.24 *μ*g/ml	NA	[31]
*Pithecellobium confertum*	Fabaceae	Medang	Seeds/methanol	NO inhibition	23.50% at 250 *μ*g/ml	NA	NA	[13]
*Portulaca oleracea*	Portulacaceae	Gelang pasir	Leaves/methanol	NO inhibition	94.80% at 250 *μ*g/ml	44 *μ*g/ml	NA	[13]
*Psophocarpus tetragonolobus*	Fabaceae	Kacang botol	Pod/methanol	Griess assay (NO inhibition)	39.28% at 100 *μ*g/ml	>100 *μ*g/ml	NA	[31]
*Sauropus androgynus*	Phyllanthaceae	Cekur manis	Leaves/methanol	Griess assay (NO inhibition)	68.28% at 100 *μ*g/ml	58.34 *μ*g/ml	NA	[31]
*Solanum nigrum*	Solanaceae	Terung meranti	Leaves/methanol	NO inhibition	27.60% at 250 *μ*g/ml	NA	NA	[13]
*Solanum torvum*	Solanaceae	Terung belanda	Leaves and fruits/methanol	NO inhibition	25.20% at 250 *μ*g/ml	NA	NA	[13]
*Thymus vulgaris*	Lamiaceae	Taim	Whole plant/methanol	LOX inhibition	62% at 100 *μ*g/ml	NA	NA	[43]
*Timonius flavescens*	Rubiaceae	Batut	Leaves/methanol	LOX inhibition	71%–100% at 100 *μ*g/ml	8.9 *μ*g/ml	NA	[26]

**Table 2:** The medicinal plants which are considered to possess anti-inflammatory activity based on *in vivo* studies.

Scientific name	Family	Local name	Part/solvent used	Dose of the extract	Experimental animals	Results	References
*Achyranthes aspera*	Amaranthaceae	Ara songsang	Root/ethyl alcohol	50, 100, and 200 mg/kg	Wistar rats	All the doses caused significant reduction in paw edema compared to control	[49]
*Annona muricata*	Annonaceae	Durian belanda	Leaves/aqueous ethanol	10–300 mg/kg	Sprague-Dawley rats	A significant decrease of the concentration of the proinflammatory cytokines TNF-*α* and IL-1*β* was observed	[50]
*Ardisia crispa*	Myrsinaceae	Mata pelandok	Root/ethanol	3, 10, 30, 100, and 300 mg/kg of body weight	Sprague-Dawley rats	A significant inhibition (93.34%) was observed in carrageenan-induced edema in rats at a dose of 300 mg/kg	[51]
*Atylosia scarabaeoides*	Fabaceae	Kara-kara/kacang kerara	Leaves/ethanol	150, 300, and 450 mg/kg	Swiss albino mice	The extract displayed significant inhibition of inflammation. Highest inhibition of paw edema (38.38%) at a dose of 450 mg/kg after 4 h of administration	[52]
*Citrullus lanatus*	Cucurbitaceae	Tembikai	Fruit pulp/petroleum ether, chloroform, and 90% ethanol	30 and 60 mg/kg of body weight	BALB/c mice	Cucurbitacin E inhibits inflammation significantly from the fourth hour and is able to revert paw edema through the COX-2 inhibition	[33]
*Corchorus capsularis*	Malvaceae	Kancing baju	Leaves/chloroform	20, 100, and 200 mg/kg	BALB/c mice and Sprague-Dawley rats	The extract caused significant reduction in the thickness of edematous paw for the first 6 h	[53]
*Crinum asiaticum*	Amaryllidaceae	Pokok bakung	Leaves/methanol	50 mg/kg of the extract	Mice	Inhibition of paw edema (94.8%)	[54]
*Curcuma aeruginosa*	Zingiberaceae	Temu hitam	Rhizomes/chloroform, methanol, and water	100, 200, 400, and 800 mg/kg	Swiss mice and Wistar rats	No significant suppression was observed after oral administration of all doses on carrageenan-induced paw edema	[55]
*Curcuma longa*	Zingiberaceae	Kunyit	Rhizomes/water	200 mg/kg of body weight	Wistar albino rats	The production of anti-inflammatory/proinflammatory cytokines is decreasing	[56]
*Cyathula prostrata*	Amaranthaceae	Ketumbar	Leaves/methanol	50,100, and 200 mg/kg	Wistar rats and Swiss albino mice	All extracts displayed a significant dose-dependent inhibition in the carrageenan-, arachidonic acid-, and xylene-induced tests	[57]
*Dicranopteris linearis*	Gleicheniaceae	Resam	Leaves/chloroform	10, 100, and 200 mg/kg	BALB/c mice and Sprague-Dawley rats	The extract produced significant anti-inflammatory activity that did not depend on the doses of the extract	[58]
*Ficus deltoidea*	Moraceae	Mas cotek	Whole plant/water	30, 100, and 300 mg/kg	Sprague-Dawley rats	The rats' paw edema volume reduced significantly in a dose-dependent manner	[59]
*Garcinia subelliptica*	Guttiferae	Pokok penanti	Seeds/chloroform	3, 10, 30, 50, and 100 *µ*M	Sprague-Dawley rats	A potent inhibitory effect on fMLP/CB-induced superoxide anion generation was observed in the isolated compound garcinielliptin oxide	[38]
*Justicia gendarussa*	Acanthaceae	Daun rusa	Root/methanol	50 mg/kg of the extract	Wistar rats	80% and 93% edema inhibition at the third and fifth hours	[60]
*Kaempferia galanga*	Zingiberaceae	Cekur	Rhizomes/chloroform	2 g/kg of the extract	Male Sprague-Dawley rats	Highest edema inhibition (42.9%)	[41]
*Manilkara zapota*	Sapotaceae	Ciku	Leaves/ethyl acetate	300 mg/kg of body weight	Albino Wistar rats	Inhibition of paw edema (92.41%)	[61]
*Mitragyna speciosa*	Rubiaceae	Biak-biak and ketom	Leaves/methanol	50, 100, and 200 mg/kg	Sprague-Dawley rats	Both doses of 100 and 200 mg/kg showed a significant inhibition of the paw edema (63%)	[62]
*Moringa oleifera*	Moringaceae	Kelur	Leaves/water	10, 30, and 100 mg/kg	BALB/c mice and Sprague-Dawley rats	Highest edema inhibition (66.7%) at the second hour at 100 mg/kg of dose	[63]
*Muntingia calabura*	Muntingiaceae	Kerukup siam	Leaves/water	27 mg/kg, 135 mg/kg, and 270 mg/kg	Sprague-Dawley rats	The extract was found to exhibit a concentration-independent anti-inflammatory activity	[64]
*Orthosiphon stamineus*	Lamiaceae	Misai kucing	Leaves/methanol : water	125, 250, 500, and 1000 mg/kg	Charles River mice and Sprague-Dawley rats	Increase in edema inhibition (26.79%)	[65]
*Peperomia pellucida*	Piperaceae	Ketumpangan air	Whole plant/petroleum ether	1000 mg/kg	Sprague-Dawley rats	The extract showed significant inhibition in magnitude of swelling after 4 h of administration	[66]
*Phyllanthus acidus*	Phyllanthaceae	Cermai	Leaves/methanol, ethyl acetate, and petroleum ether	250 and 500 mg/kg	Wistar rats and albino mice	All the extracts showed reduction in carrageenan-induced paw edema with highest inhibition (90.91%) in the methanol extract	[67]
*Physalis minima*	Solanaceae	Pokok letup-letup	Whole plant/methanol and chloroform fraction	200 and 400 mg/kg	NMRI mice and Wistar rats	Crude extract and chloroform fraction showed highest inhibition of paw edema at 66% and 68% at 400 mg/kg, respectively	[68]
*Piper sarmentosum*	Piperaceae	Kaduk	Leaves/water	30–300 mg/kg of the extract	Sprague-Dawley rats and male BALB/c mice	All doses exerted anti-inflammatory activity in a dose-dependent manner	[69]
*Polygonum minus*	Polygonaceae	Kesum	Aerial parts/water	100 mg/kg and 300 mg/kg	Wistar albino rats	The extracts significantly reduced the paw edema volume in the rats after 4 h	[70]
*Sandoricum koetjape*	Meliaceae	Sentul	Stems/methanol	5 mg/ear	BALB/c mice	A significant inhibition (94%) in TPA-induced edema was observed in the isolated compound 3-oxo-12-oleanen-29-oic acid	[71]
*Solanum nigrum*	Solanaceae	Terung meranti	Leaves/water	10, 50, and 100% of concentration	BALB/c mice and Sprague-Dawley rats	Extracts produce apparently two-phase anti-inflammatory activity: the first phase between 1 and 2 h and the second phase between 5 and 7 h after carrageenan administration	[72]
*Stachytarpheta jamaicensis*	Verbenaceae	Selasih dandi	Leaves/ethanol	50, 100, and 150 mg/kg	BALB/c albino strain mice and Sprague-Dawley rats	A significant dose-dependent anti-inflammatory activity was observed 30 min after the administration of the extract at all doses	[73]
*Vitex negundo*	Lamiaceae	Legundi	Leaves/ethanol	2 mg/ear	Mice	The extract showed an inhibitory activity of 54.1%	[74]
*Zingiber zerumbet*	Zingiberaceae	Lempoyang	Rhizomes/methanol	25, 50, and 100 mg/kg	BALB/c mice	A significant antiedema activity was observed at all doses in a dose-dependent manner (i.e., 50 and 100 mg/kg doses of the extract exhibited activity at 90 min after administration, while 25 mg/kg exhibited at 150 min)	[75]
				5, 10, 50, and 100 mg/kg	ICR mice	The isolated compound (zerumbone) significantly showed dose-dependent inhibition of paw edema induced by carrageenan at all doses (5, 10, 50, and 100 mg/kg) in mice with percentage of inhibition of 33.3, 66.7, 83.3, and 83.3%, respectively	[76]

## References

[1] Lee C. H., Choi E. Y. (2018). Macrophages and inflammation. *Journal of Rheumatic Diseases*.

[2] Rainsford K. D. (2007). Anti-inflammatory drugs in the 21st century. *Inflammation in the Pathogenesis of Chronic Diseases*.

[3] Yedgar S., Krimsky M., Cohen Y., Flower R. J. (2007). Treatment of inflammatory diseases by selective eicosanoid inhibition: a double-edged sword?. *Trends in Pharmacological Sciences*.

[4] Monnier Y., Zaric J., Ruegg C. (2005). Inhibition of angiogenesis by non-steroidal anti-inflammatory drugs: from the bench to the bedside and back. *Current Drug Targets-Inflammation and Allergy*.

[5] Fuster V., Sanz J. (2007). Vascular inflammation. *Journal of the American Society of Hypertension*.

[6] McKellar G., Madhok R., Singh G. (2007). The problem with NSAIDs: what data to believe?. *Current Pain and Headache Reports*.

[7] Akkol E. K. (2012). New strategies for anti-inflammatory drug development. *Journal of Pharmacogenomics and Pharmacoproteomics*.

[8] Russo D., Valentão P., Andrade P. B., Fernandez E. C., Milella L. (2015). Evaluation of antioxidant, antidiabetic and anticholinesterase activities of *Smallanthus sonchifolius* landraces and correlation with their phytochemical profiles. *International Journal of Molecular Sciences*.

[9] Wirth J. H., Hudgins J. C., Paice J. A. (2005). Use of herbal therapies to relieve pain: a review of efficacy and adverse effects. *Pain Management Nursing*.

[10] Salleh M. N., Kadir A. A., Shaari K., Ibrahim Z., Zakri A. H. (1995). Medicinal products from the tropical rain forests of the Far East 95-105. *Prospects in Bodiversity Prospecting*.

[11] Latif A. Medicinal and aromatic plants of Asia: approaches to exploitation and conservation.

[12] Abu Bakar F. I., Abu Bakar M. F., Rahmat A., Abdullah N., Sabran S. F., Endrini S. (2018). Anti-gout potential of Malaysian medicinal plants. *Frontiers in Pharmacology*.

[13] Abas F., Lajis N. H., Israf D. A., Khozirah S., Kalsom Y. U. (2006). Antioxidant and nitric oxide inhibition activities of selected Malay traditional vegetables. *Food Chemistry*.

[14] WHO (World Health Organization) (2011). The world traditional medicines situation. *Traditional Medicines: Global Situation, Issues and Challenges*.

[15] De Luca V., Salim V., Atsumi S. M., Yu F. (2012). Mining the biodiversity of plants: a revolution in the making. *Science*.

[16] Burkill I. H. (1966). *A Dictionary of the Economic Products of the Malay Peninsula*.

[17] Kulip J., Fan L. N., Manshoor N. (2010). Medicinal plants in Maliau Basin, Sabah, Malaysia. *Journal of Tropical Biology and Conservation*.

[18] Coussens L. M., Werb Z. (2002). Inflammation and cancer. *Nature*.

[19] Zamora R., Vodovotz Y., Billiar T. R. (2000). Inducible nitric oxide synthase and inflammatory diseases. *Molecular Medicine*.

[20] Wright H. L., Moots R. J., Bucknall R. C., Edwards S. W. (2010). Neutrophil function in inflammation and inflammatory diseases. *Rheumatology*.

[21] Kumar V., Abbas A. K., Fausto N., Aster J. C. (2014). Robbins and Cotran pathologic basis of disease. *Professional Edition E-Book*.

[22] Kanterman J., Sade-Feldman M., Baniyash M. (2012). New insights into chronic inflammation-induced immunosuppression. *Seminars in Cancer Biology*.

[23] Kundu J. K., Surh Y. J. (2008). Inflammation: gearing the journey to cancer. *Mutation Research/Reviews in Mutation Research*.

[24] Kundu J. K., Surh Y. J. (2012). Emerging avenues linking inflammation and cancer. *Free Radical Biology and Medicine*.

[25] Burdan F., Szumilo J., Klepacz R. (2004). Gastrointestinal and hepatic toxicity of selective and non-selective cyclooxygenase-2 inhibitors in pregnant and non-pregnant rats. *Pharmacological Research*.

[26] Chung L. Y., Soo W. K., Chan K. Y., Mustafa M. R., Goh S. H., Imiyabir Z. (2009). Lipoxygenase inhibiting activity of some Malaysian plants. *Pharmaceutical Biology*.

[27] Behbahani M., Ali A. M., Muse R., Mohd N. B. (2007). Anti-oxidant and anti-inflammatory activities of leaves of *Barringtonia racemosa*. *Journal of Medicinal Plants Research*.

[28] Isa N. M., Abdelwahab S. I., Mohan S. (2012). *In vitro* anti-inflammatory, cytotoxic and antioxidant activities of boesenbergin A, a chalcone isolated from *Boesenbergia rotunda* (L.) (fingerroot). *Brazilian Journal of Medical and Biological Research*.

[29] Afsar V., Reddy Y. M., Saritha K. V. (2012). *In vitro* antioxidant activity and anti-inflammatory activity of methanolic leaf extract of *Boswellia serrata*. *International Journal of Life Sciences Biotechnology and Pharma Research*.

[30] Mogana R., Teng-Jin K., Wiart C. (2013). Anti-inflammatory, anticholinesterase, and antioxidant potential of scopoletin isolated from *Canarium patentinervium* Miq. (Burseraceae Kunth). *Evidence-Based Complementary and Alternative Medicine*.

[31] Lee K. H., Padzil A. M., Syahida A. (2011). Evaluation of anti-inflammatory, antioxidant and anti-nociceptive activities of six Malaysian medicinal plants. *Journal of Medicinal Plants Research*.

[32] Chan K. Y., Mohamad K., Ooi A. J., Imiyabir Z., Chung L. Y. (2012). Bioactivity-guided fractionation of the lipoxygenase and cyclooxygenase inhibiting constituents from *Chisocheton polyandrus* Merr.. *Fitoterapia*.

[33] Abdelwahab S. I., Hassan L. E. A., Sirat H. M. (2011). Anti-inflammatory activities of cucurbitacin E isolated from *Citrullus lanatus* var. citroides: role of reactive nitrogen species and cyclooxygenase enzyme inhibition. *Fitoterapia*.

[34] Kim Y. H., Kim K. H., Han C. S. (2007). Anti-inflammatory activity of *Crinum asiaticum* Linne var. japonicum extract and its application as a cosmeceutical ingredient. *Journal of Cosmetic Science*.

[35] Ramsewak R. S., DeWitt D. L., Nair M. G. (2000). Cytotoxicity, antioxidant and anti-inflammatory activities of curcumins I–III from *Curcuma longa*. *Phytomedicine*.

[36] Varghese C. P., Ambrose C., Jin S. C., Lim Y. J., Keisaban T. (2013). Antioxidant and anti-inflammatory activity of *Eurycoma longifolia* Jack, a traditional medicinal plant in Malaysia. *International Journal of Pharmaceutical Sciences and Nanotechnology*.

[37] Abdullah Z., Hussain K., Ismail Z., Ali R. M. (2009). Anti-inflammatory activity of standardised extracts of leaves of three varieties of *Ficus deltoidea*. *International Journal of Pharmaceutical and Clinical Research*.

[38] Weng J. R., Lin C. N., Tsao L. T., Wang J. P. (2003). Novel and anti-inflammatory constituents of *Garcinia subelliptica*. *Chemistry-A European Journal*.

[39] Siriwatanametanon N., Fiebich B. L., Efferth T., Prieto J. M., Heinrich M. (2010). Traditionally used Thai medicinal plants: in vitro anti-inflammatory, anticancer and antioxidant activities. *Journal of Ethnopharmacology*.

[40] Oskoueian E., Abdullah N., Saad W. Z. (2011). Antioxidant, anti-inflammatory and anticancer activities of methanolic extracts from *Jatropha curcas* Linn. *Journal of Medicinal Plants Research*.

[41] Umar M. I., Asmawi M. Z., Sadikun A. (2012). Bioactivity-guided isolation of ethyl-*p*-methoxycinnamate, an anti-inflammatory constituent, from *Kaempferia galanga* L. extracts. *Molecules*.

[42] Karimi E., Jaafar H. Z., Ahmad S. (2013). Antifungal, anti-inflammatory and cytotoxicity activities of three varieties of *Labisia pumila* benth: from microwave obtained extracts. *BMC Complementary and Alternative Medicine*.

[43] Beidokhti M. N., Prakash H. S. (2013). Antioxidant and anti-inflammatory potential of selected medicinal plants of Lamiaceae family. *International Journal of Pharmacy and Pharmaceutical Sciences*.

[44] Kutoi C. J., Yen K. H., Seruji N. M. U. Pharmacology evaluation of *Litsea garciae* (Lauraceae).

[45] Shaari K., Suppaiah V., Wai L. K. (2011). Bioassay-guided identification of an anti-inflammatory prenylated acylphloroglucinol from *Melicope ptelefolia* and molecular insights into its interaction with 5-lipoxygenase. *Bioorganic and Medicinal Chemistry*.

[46] Cheenpracha S., Park E. J., Yoshida W. Y. (2010). Potential anti-inflammatory phenolic glycosides from the medicinal plant *Moringa oleifera* fruits. *Bioorganic and Medicinal Chemistry*.

[47] Laavola M., Nieminen R., Yam M. F. (2012). Flavonoids eupatorin and sinensetin present in *Orthosiphon stamineus* leaves inhibit inflammatory gene expression and STAT1 activation. *Planta Medica*.

[48] Hendra R., Ahmad S., Oskoueian E., Sukari A., Shukor M. Y. (2011). Antioxidant, anti-inflammatory and cytotoxicity of *Phaleria macrocarpa* (Boerl.) Scheff Fruit. *BMC Complementary and Alternative Medicine*.

[49] Kumar S. V., Sankar P., Varatharajan R. (2009). Anti-inflammatory activity of roots of *Achyranthes aspera*. *Pharmaceutical Biology*.

[50] Foong C. P., Hamid R. A. (2012). Evaluation of anti-inflammatory activities of ethanolic extract of *Annona muricata* leaves. *Revista Brasileira de Farmacognosia*.

[51] Roslida A., Kim K. (2008). Anti-inflammatory and anti-hyperalgesic effects of *Ardisia crispa* Thunb. D.C. *Pharmacognosy Magazine*.

[52] Sarwar S., Sufian M. A., Islam M. R. (2017). Investigation of *in vivo* analgesic, anti-inflammatory, *in vitro* membrane stabilizing and thrombolytic activities of *Atylosia scarabaeoides* and *Crotalaria spectabilis* leaves. *Journal of Pharmacology and Toxicology*.

[53] Zakaria Z. A., Sulaiman M. R., Gopalan H. K. (2007). Antinociceptive and anti-inflammatory properties of *Corchorus capsularis* leaves chloroform extract in experimental animal models. *Yakugaku Zasshi*.

[54] Samud A. M., Asmawi M. Z., Sharma J. N., Yusof A. P. M. (1999). Anti-inflammatory activity of *Crinum asiaticum* plant and its effect on bradykinin-induced contractions on isolated uterus. *Immunopharmacology*.

[55] Reanmongkol W., Subhadhirasakul S., Khaisombat N., Fuengnawakit P., Jantasila S., Khamjun A. (2006). Investigation the antinociceptive, antipyretic and anti-inflammatory activities of *Curcuma aeruginosa* Roxb. extracts in experimental animals. *Journal of Science Technology*.

[56] Ramadan G., Al-Kahtani M. A., El-Sayed W. M. (2011). Anti-inflammatory and anti-oxidant properties of *Curcuma longa* (turmeric) versus *Zingiber officinale* (ginger) rhizomes in rat adjuvant-induced arthritis. *Inflammation*.

[57] Ibrahim B., Sowemimo A., van Rooyen A., Van de Venter M. (2012). Antiinflammatory, analgesic and antioxidant activities of *Cyathula prostrata* (Linn.) Blume (Amaranthaceae). *Journal of Ethnopharmacology*.

[58] Zakaria Z. A., Ghani Z. D. F. A., Nor R. N. S. R. M., Gopalan H. K., Sulaiman M. R., Abdullah F. C. (2006). Antinociceptive and anti-inflammatory activities of *Dicranopteris linearis* leaves chloroform extract in experimental animals. *Yakugaku Zasshi*.

[59] Zakaria Z. A., Hussain M. K., Mohamad A. S., Abdullah F. C., Sulaiman M. R. (2012). Anti-inflammatory activity of the aqueous extract of *Ficus deltoidea*. *Biological Research for Nursing*.

[60] Kumar K. S., Vijayan V., Bhaskar S., Krishnan K., Shalini V., Helen A. (2012). Anti-inflammatory potential of an ethyl acetate fraction isolated from *Justicia gendarussa* roots through inhibition of iNOS and COX-2 expression via NF-κB pathway. *Cellular Immunology*.

[61] Ganguly A., Al Mahmud Z., Uddin M. M. N., Rahman S. A. (2013). *In-vivo* anti-inflammatory and anti-pyretic activities of *Manilkara zapota* leaves in albino Wistar rats. *Asian Pacific Journal of Tropical Disease*.

[62] Mossadeq W. S., Sulaiman M. R., Mohamad T. T. (2009). Anti-inflammatory and antinociceptive effects of *Mitragyna speciosa* Korth methanolic extract. *Medical Principles and Practice*.

[63] Sulaiman M. R., Zakaria Z. A., Bujarimin A. S., Somchit M. N., Israf D. A., Moin S. (2008). Evaluation of *Moringa oleifera* aqueous extract for antinociceptive and anti-inflammatory activities in animal models. *Pharmaceutical Biology*.

[64] Zakaria Z. A., Hazalin N. M. N., Zaid S. M. (2007). Antinociceptive, anti-inflammatory and antipyretic effects of *Muntingia calabura* aqueous extract in animal models. *Journal of Natural Medicines*.

[65] Yam M. F., Asmawi M. Z., Basir R. (2008). An investigation of the anti-inflammatory and analgesic effects of *Orthosiphon stamineus* leaf extract. *Journal of Medicinal Food*.

[66] Mutee A. F., Salhimi S. M., Yam M. F. (2010). *In vivo* anti-inflammatory and *in vitro* antioxidant activities of *Peperomia pellucida*. *International Journal of Pharmacology*.

[67] Chakraborty R., Biplab D., Devanna N., Sen S. (2012). Antiinflammatory, antinociceptive and antioxidant activities of *Phyllanthus acidus* L. extracts. *Asian Pacific Journal of Tropical Biomedicine*.

[68] Khan M. A., Khan H., Khan S., Mahmood T., Khan P. M., Jabar A. (2009). Anti-inflammatory, analgesic and antipyretic activities of *Physalis minima* Linn. *Journal of Enzyme Inhibition and Medicinal Chemistry*.

[69] Zakaria Z. A., Mohamad A. S., Chear C. T., Wong Y. Y., Israf D. A., Sulaiman M. R. (2010). Antiinflammatory and antinociceptive activities of *Zingiber zerumbet* methanol extract in experimental model systems. *Medical Principles and Practice*.

[70] George A., Chinnappan S., Chintamaneni M. (2014). Anti-inflammatory effects of *Polygonum minus* (Huds) extract (Lineminus™) in *in-vitro* enzyme assays and carrageenan induced paw edema. *BMC Complementary and Alternative Medicine*.

[71] Rasadah M. A., Khozirah S., Aznie A. A., Nik M. M. (2004). Anti-inflammatory agents from *Sandoricum koetjape* Merr. *Phytomedicine*.

[72] Zakaria Z. A., Sulaiman M. R., Morsid N. A. (2009). Antinociceptive, anti-inflammatory and antipyretic effects of *Solanum nigrum* aqueous extract in animal models. *Methods and Findings in Experimental and Clinical Pharmacology*.

[73] Sulaiman M. R., Zakaria Z. A., Chiong H. S., Lai S. K., Israf D. A., Shah T. A. (2009). Antinociceptive and anti-inflammatory effects of *Stachytarpheta jamaicensis* (L.) Vahl (Verbenaceae) in experimental animal models. *Medical Principles and Practice*.

[74] Yunos N. M., Mat Ali R., Kean O. B., Abas R. (2005). Cytotoxicity evaluations on *Vitex negundo* anti-inflammatory extracts. *Malaysian Journal of Science*.

[75] Zakaria Z. A., Patahuddin H., Mohamad A. S., Israf D. A., Sulaiman M. R. (2010). *In vivo* anti-nociceptive and anti-inflammatory activities of the aqueous extract of the leaves of *Piper sarmentosum*. *Journal of Ethnopharmacology*.

[76] Sulaiman M. R., Perimal E. K., Akhtar M. N. (2010). Anti-inflammatory effect of zerumbone on acute and chronic inflammation models in mice. *Fitoterapia*.

[77] Pacher P., Beckman J. S., Liaudet L. (2007). Nitric oxide and peroxynitrite in health and disease. *Physiological Reviews*.

[78] Fukumura D., Gohongi T., Kadambi A. (2001). Predominant role of endothelial nitric oxide synthase in vascular endothelial growth factor-induced angiogenesis and vascular permeability. *Proceedings of the National Academy of Sciences*.

[79] Kleinert H., Pautz A., Linker K., Schwarz P. M. (2004). Regulation of the expression of inducible nitric oxide synthase. *European Journal of Pharmacology*.

[80] De Caterina R., Zampolli A. (2004). From asthma to atherosclerosis—5-lipoxygenase, leukotrienes, and inflammation. *New England Journal of Medicine*.

[81] Zhang Y. Y., Walker J. L., Huang A. (2002). Expression of 5-lipoxygenase in pulmonary artery endothelial cells. *Biochemical Journal*.

[82] Schmidt C., Kosche E., Baumeister B., Vetter H. (1995). Arachidonic acid metabolism and intracellular calcium concentration in inflammatory bowel disease. *European Journal of Gastroenterology and Hepatology*.

[83] Chen M., Lam B. K., Kanaoka Y. (2006). Neutrophil-derived leukotriene B4 is required for inflammatory arthritis. *Journal of Experimental Medicine*.

[84] Rubin P., Mollison K. W. (2007). Pharmacotherapy of diseases mediated by 5-lipoxygenase pathway eicosanoids. *Prostaglandins and Other Lipid Mediators*.

[85] Kwon O. S., Choi J. S., Islam M. N., Kim Y. S., Kim H. P. (2011). Inhibition of 5-lipoxygenase and skin inflammation by the aerial parts of *Artemisia capillaris* and its constituents. *Archives of Pharmacal Research*.

[86] Vane J. R., Botting R. M. (1996). Mechanism of action of anti-inflammatory drugs. *Scandinavian Journal of Rheumatology*.

[87] Ryseck R. P., Raynoschek C., Macdonald-Bravo H., Dorfman K., Mattei M. G., Bravo R. (1992). Identification of an immediate early gene, pghs-B, whose protein product has prostaglandin synthase/cyclooxygenase activity. *Cell Growth and Differentiation*.

[88] Mohan M., Gulecha V. S., Aurangabadkar V. M., Balaraman R., Austin A., Thirungnanasampathan S. (2009). Analgesic and anti-inflammatory activity of a polyherbal formulation (PHF-AROGH). *Oriental Pharmacy and Experimental Medicine*.

[89] Panthong A., Norkaew P., Kanjanapothi D., Taesotikul T., Anantachoke N., Reutrakul V. (2007). Anti-inflammatory, analgesic and antipyretic activities of the extract of gamboge from *Garcinia hanburyi* Hook f.. *Journal of Ethnopharmacology*.

[90] Crunkhorn P., Meacock S. C. R. (1971). Mediators of the inflammation induced in the rat paw by carrageenin. *British Journal of Pharmacology*.

[91] Van Arman C. G., Begany A. J., Miller L. M., Pless H. H. (1965). Some details of the inflammations caused by yeast and carrageenin (with appendix on kinetics of the reaction). *Journal of Pharmacology and Experimental Therapeutics*.

[92] Brooks P. M., Day R. O. (1991). Nonsteroidal antiinflammatory drugs—differences and similarities. *New England Journal of Medicine*.

[93] Abad T., McNaughton-Smith G., Fletcher W. Q. (2000). Isolation of (S)-(+)-naproxene from *Musa acuminata*. Inhibitory effect of naproxene and its 7-methoxy isomer on constitutive COX-1 and inducible COX-2. *Planta Medica*.

[94] Bissonnette E. Y., Tremblay G. M., Turmel V., Pirotte B., Reboud-Ravaux M. (2009). Coumarinic derivatives show anti-inflammatory effects on alveolar macrophages, but their anti-elastase activity is essential to reduce lung inflammation *in vivo*. *International Immunopharmacology*.

[95] Pendzhiev A. M. (2002). Proteolytic enzymes of papaya: medicinal applications. *Pharmaceutical Chemistry Journal*.

[96] Gonzalez-Gallego J., Sánchez-Campos S., Tunon M. J. (2007). Anti-inflammatory properties of dietary flavonoids. *Nutricion Hospitalaria*.

